# Huge Dose Vitamin B12 (Vit B12) Treatment for Pernicious Anemia

**DOI:** 10.4274/Tjh.2013.0053

**Published:** 2013-06-05

**Authors:** Şinasi Özsoylu

**Affiliations:** 1 Fatih University School of Medicine, Department of Hematology, Ankara, Turkey

## TO THE EDITOR

The recent letter by Aytaç and colleagues, entitled “Poland syndrome associated with pernicious anemia and gastric dysplasia”, gives me an opportunity to question the vitamin B12 dose administered for the treatment of pernicious anemia [1].

Vitamin B12 was initiated by the authors (1000 µg daily for 5 days, 1000 µg weekly for 4 weeks, and then 1000 µg monthly for life) without taking into account that its daily requirement is 2 µg and its half-life is more than 350 days [2,3,4,5].

Although to my knowledge, vitamin B12 toxicity has not been reported, its extra benefit related to huge doses is also not known.

On this occasion, I would like to bring to attention that the Poland syndrome’s association with leukemia was previously reported by us [6].

## AUTHORS' REPLY: B-12 REGIMEN FOR TREATMENT OF PERNICIOUS ANEMIA

It has been a great honor for us to have a chance to criticize the debatable topic with Professor Özsoylu, we appreciate his kind interest to our case presentation and also giving us the opportunity to comment on this issue. The management strategy of the adult patient in our report was planned by considering existing B-12 deficiency and the operation performed subsequently, a total gastrectomy. Although daily requirement and in-vivo metabolism of B-12 is known, some of the expert hematologists treat pernicious anemia with high dose B-12 in current clinical practice [7,8]. They also claim that beginning with high dose B-12 replacement helps delay relapse especially in patients who may discontinue treatment [7]. While there are various recommended schedules for vitamin B12 injections, there is no consensus on an exact B-12 regimen for pernicious anemia [8]. As Professor Özsoylu stated, new studies questioning B-12 regimen for pernicious anemia are needed.

**Erman Aytac, Deram Buyuktas**
